# Theranostics on the immunoactivity of T cells

**DOI:** 10.1002/ctm2.1421

**Published:** 2023-09-15

**Authors:** Changrong Shi, Xinyi Zhang, Xiaomin Liu, Xiaoyuan Chen, Zijian Zhou

**Affiliations:** ^1^ State Key Laboratory of Infectious Disease Vaccine Development Xiang An Biomedicine Laboratory & Center for Molecular Imaging and Translational Medicine School of Public Health, Shenzhen Research Institute of Xiamen University Xiamen University Xiamen China; ^2^ Departments of Diagnostic Radiology Surgery, Chemical and Biomolecular Engineering Biomedical Engineering Yong Loo Lin School of Medicine and College of Design and Engineering National University of Singapore Singapore Singapore; ^3^ Clinical Imaging Research Centre Centre for Translational Medicine Yong Loo Lin School of Medicine National University of Singapore Singapore Singapore; ^4^ Nanomedicine Translational Research Program Yong Loo Lin School of Medicine National University of Singapore Singapore Singapore; ^5^ Institute of Molecular and Cell Biology Agency for Science, Technology, and Research (A*STAR) Singapore Singapore

## 1 INTRODUCTION

The immuoactivation and the ensuing therapeutic outcomes greatly rely on the performance of T cells in the tumours. However, a pitfall was that the immune‐activated T cells would suffer from the loss of activity in the tumour microenvironment, resulting in compromised therapeutic efficacy. This phenomenon is known as T‐cell exhaustion which is governed by multiple mechanisms (e.g., antigen exposure, metabolism). A clinical study showed that the percentage of intratumorally CD8^+^ T cells that recognise the autologous tumour cells could be as low as only ∼10%.^1^, ^2^ It is thus critical to distil the in situ activity rather than looking at the population of T cells in the tumours during the immunotherapy. From a vantage point of chemical biology, the redox status orchestrates the energetic metabolism of most aerobic organisms, which shed light on the immunomodulation and activation of T cells. To this end, there is an open question against how to regulate and quantify the in situ activity of T cells in the tumour? In our recent work, we showed that targeting the redox metabolism of T cell by chemical approaches could realise efficient modulation and molecular imaging of the immune response in the treatment of cancer models.

## 2 CHALLENGES ON THERANOSTICS OF T‐CELL IMMUNOACTIVITY

### 2.1 To regulate the T‐cell activity in the tumour microenvironment

Various approaches have been explored to overcome T‐cell exhaustion and enhance the activity of T cells in the tumours. One strategy is to use immune checkpoint inhibitors, which block the inhibitory pathways and release the brakes on the activity of T cells.^3^ This strategy allows T cells to recover the functions to combat cancer cells. Despite these important advances, only certain subsets of T cells could be reinvigorated.^4^ There are many other factors, such as, resistance of targeted cells, metabolic status, hypoxia, etc., may also affect the tempo of exhaustion.^5^ Other methods include adoptive T‐cell therapy, in which T cells are extracted from the patient, modified, expanded in vitro, and then infused back into the patient to boost their antitumour capabilities.^6^, ^7^ Unfortunately, the broad effectiveness of T cell‐based cancer therapy in the clinic is still elusive. Most representative adoptive T‐cell products for solid tumour types exhibit low durable response rates.^8^, ^9^ The regulation of T‐cell activity in the tumour microenvironment remains a challenge to improve T cell‐mediated immunotherapy.

### 2.2 To quantify the T‐cell activity in the tumours

The population of T cells within tumours may not be an accurate indicator of their functional activity. Hence, a better understanding of T‐cell activity will help clinicians tailoring the immunotherapy in an effective manner. Tissue biopsy is the traditional gold standard for studying the pathology in the tumours, which involves an invasive sampling process.^10^ Nevertheless, due to the spatial and temporal heterogeneity of tumour tissues, there are certain levels of discrepancy between the biopsy results and the overall perspectives of the tumours. To overcome these limitations, efforts have been made to identify new biomarkers and develop molecular imaging techniques for noninvasive analysis of T cells in the tumour. The isotopes (e.g., ^89^Zr, ^64^Cu)‐labelled antibodies (e.g., anti‐CD3, anti‐CD4, and anti‐CD8 Abs) have been used to track respective T‐cell populations in response to immunotherapy by positron emission tomograph.^11^, ^12^ These strategies provide invaluable prognostic information for improved therapeutic outcomes. However, the method of imaging the cell surface makers alone may come to unconcluded information on immune status. Immune response analysis without characterising the activity of T cell may lead to underestimation of the immune status.^13^ Thus, molecular imaging approaches enabling to delineate the in situ immune activity of T cells in the tumour remains a formidable challenge.

## 3 THERANOSTICS ON T‐CELL IMMUNOACTIVITY IN SOLID TUMOURS

We reported a chemical biology approach to modulate and quantitatively image the activity of T cells by magnetic resonance imaging (MRI).^14^ In this work, we designed T‐cell targeted fusogenic liposomes (T‐Fulips) composed of CD3 targeting fragments and reactive oxygen species (ROS) scavenging molecules. The T‐Fulips on the membrane surface of T cells serve as ROS ‘decoys’ in the extracellular milieu, harbouring T cells from oxidation‐induced loss of activity. Meanwhile, the process of ROS scavenging leads to changes in molecular magnetism which could be quantitatively measured by the change of relaxation time in MRI. In this way, we were able to regulate and stratify the T‐cell based immune response at multiple mouse tumour models. The success of engineering T cell surface redox status for cancer theranostics hinged on two crucial findings. First, an increased number of reduced thiol groups (‐SH) on the membrane surface of T cells, governed by the reduced form of thioredoxin (Trx) and surface molecules (e.g., CD4), is associated with the enhanced activity of T cells, and vice versa.^15^ This finding emphasised the significance of targeting the redox status on the membrane surface rather than employing an intracellular delivery approach. Second, the TEMP groups on T‐Fulips act as ‘decoys’ to scavenge ROS and to protect T cells from ROS‐induced oxidation of ‐SH groups and the resulted loss of activity. The use of TEMP groups to scavenge ROS concomitantly activated MRI contrast enhancement due to the ROS‐induced production of paramagnetic TEMPO groups. By centring the ‘0’ to ‘1’ activation of the contrast enhancement in MRI from diamagnetic TEMP to paramagnetic TEMPO transformation, this method effectively stratified the T‐cell activity with high sensitivity and reliability. The exploration of redox balance in T cells offers a fascinating opportunity for modulation and molecular imaging of the immune response in immunotherapy.

In the past decade, adoptive T‐cell therapy has been a revolutionary approach in the treatment of specific blood cancers, such as leukaemia and lymphoma. This therapy involves extracting T cells from a patient, modifying them to boost their cancer‐fighting abilities, and then reintroducing them back into the patient to target and destroy cancer cells. However, the efficacy of adoptive T‐cell therapy in solid tumours has been restrained due to the presence of physical barriers and biochemical factors in the tumour microenvironment, which poses great challenges to the infiltration and functionalisation of T cells.^16^ In a B16F10‐OVA model, we demonstrated that the T‐Fulips could enhance the therapeutic efficacy of adoptive T cell therapy. Though the results based on the regulatory role of the T‐Fulips have yet to be widely reported in other solid tumour models, our approach exemplifies the potential of targeting the redox metabolism by chemical approaches to manage the activity of adoptive T cells. One drawback of T‐Fulips is that their ‘decoys’ scavenging ROS will be diminished after a certain period of T‐cell activation. Fortunately, T‐Fulips can be further derivatised to include a variety of antioxidants like vitamin C and astaxanthin, and nano‐enzymes such as cerium oxide, iron oxide. These agents protect T cells from the elevated level of ROS, thus allowing adoptive T‐cell therapy to trigger strong immune responses against solid tumours. Currently, efforts are being made to arm T cells with antitumour activity in patients hospitalised with solid tumours, ensuring that such treatments remain effective and beneficial in the long run. For example, the collaboration with clinicians to encompass additional solid tumour types is necessary, such as advanced liver cancers and breast cancers. In addition, new strategies to mitigate or interrupt adoptive T‐cell exhaustion in the tumours for improving treatment outcomes are also meaningful, such as synthesising new antioxidant adjuvant and regulating redox metabolism genes.

## 4 FUTURE OUTLOOK

**FIGURE 1:**
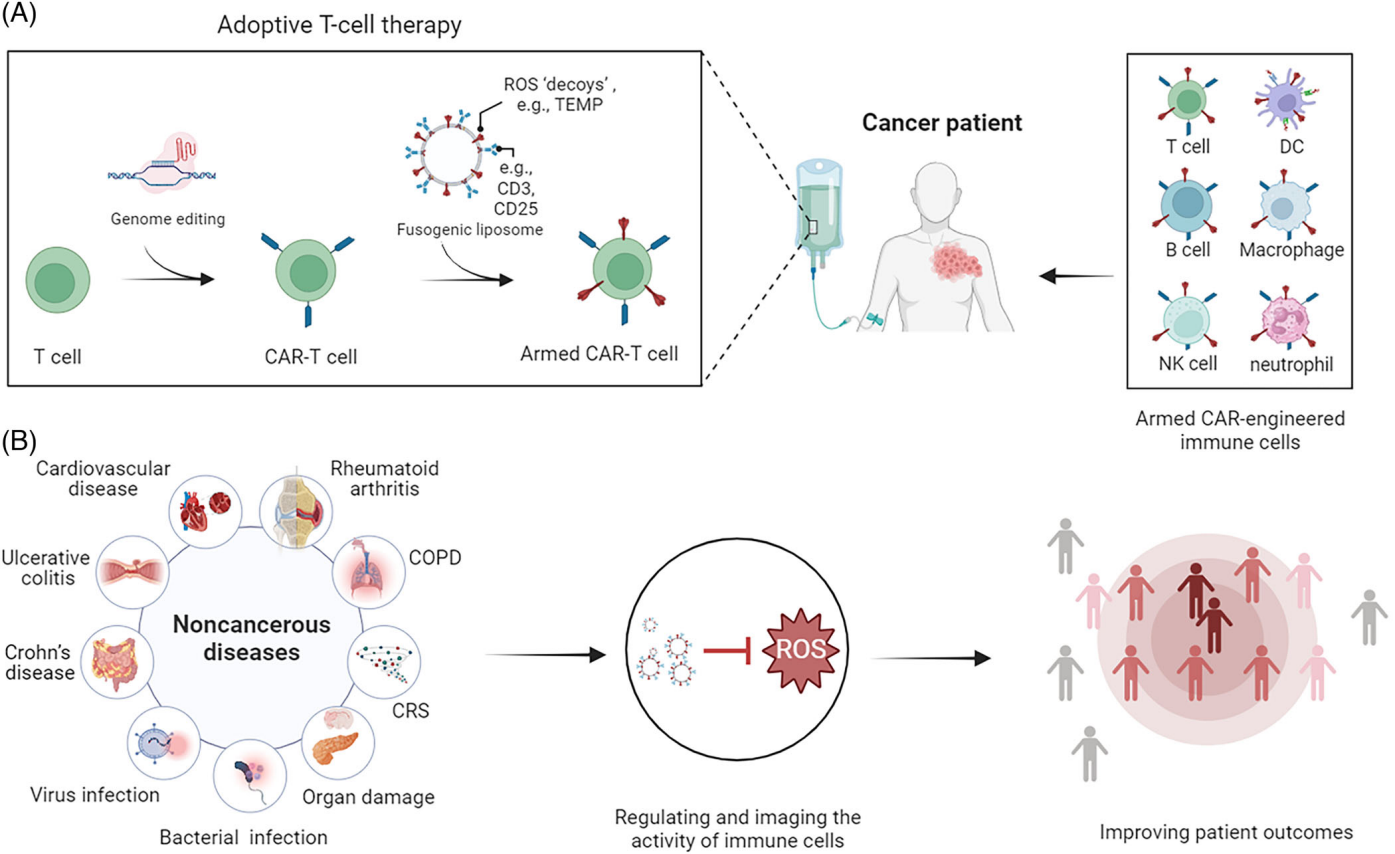
The potential value of targeting the redox metabolism by chemical approaches in the treatment of cancer patients and noncancerous diseases. (A) Comparing with the traditional process of preparing chimeric antigen receptor (CAR) engineered T cell (CAR‐T) based on the gene editing method, a novel adoptive immune‐cell therapy is proposed to engineering immune cells (e.g., T cell, B cell, NK cell, neutrophils, macrophage, and DC) to contain ROS ‘decoys’ (e.g., TEMP group) on the membrane surface. These ‘decoys’ could scavenge ROS, thereby protecting immune cells from ROS‐induced inactivation. The diversification of fusogenic liposomes, including different targets (e.g., CD3, CD25), provides an exceptional means of targeting a wide range of immune cells. (B) The strategy of targeting the redox balance in T cells could be applicable to noncancerous diseases to the benefit of patient outcomes, such as cardiovascular disease, rheumatoid arthritis, COPD, organ damage complications, virus infection, bacterial infection, ulcerative colitis, and Crohn's disease, etc.

Maintaining the redox balance in the microenvironment is crucial for ensuring proper immune cell responses and the overall immune functions.^17^ By demonstrating the benefit of T cells armed with T‐Fulips, our results indicated the potential value of targeting the redox metabolism by chemical approaches in the clinical treatment of cancer patients (Figure 1A). Theoretically, this strategy could also be applied to other immune cells, including B cells, natural killer cells (NKs), neutrophils, macrophages, and dendritic cells, to similarly achieve therapeutic goals. Emerging clinical and preclinical evidence indicated that these immune cells also showed sensitivity to changes of the redox environment.^18^, ^19^ Furthermore, the optimal time and dose to cancer treatments are likely to vary depending on the pathogenesis of the different tumour microenvironment. Functional imaging of the activity of immune cells based on targeting the redox metabolism may expand our ability to tailor individualised treatment. Ultimately, this approach may allow us to determine the most appropriate therapy for the right cancer patient at the right time, thus highlighting the potential of personalised cancer treatments.

The exploration of redox balance in T cells also provokes broad interest to noncancerous diseases (Figure 1B). Mounting evidence highlights that antioxidant‐based drugs have shown encouraging therapeutic efficacy in treating noncancerous diseases, such as chronic inflammatory diseases, rheumatoid arthritis, virus infection (for example, COVID‐19), cardiovascular disease, and organ damage complications in both clinical and preclinical studies.^20^, ^21^ For rheumatoid arthritis, antioxidant agents like vitamin C, vitamin E, and selenium have shown promise in alleviating the inflammation and joint pain.^22^ Similarly, in inflammatory bowel diseases like ulcerative colitis and Crohn's disease, antioxidants have been investigated for their ability to ameliorate intestinal inflammation and to improve the symptoms.^23^ Respiratory diseases such as asthma and chronic obstructive pulmonary disease (COPD) have displayed reduced airway inflammation and bronchoconstriction due to antioxidant therapy which lower the inflammatory responses and cellular damage through reducing the oxidative stress.^24^ The benefit of antioxidant‐based drugs to a wide range of different pathologies suggests that regulating the redox balance in immune cells may have broad applications. The current results in cancer therapy could thus be extended to manage noncancerous diseases as well. We believe that the understanding of the characterisation of the redox balance during immune responses could help to develop new imaging method that allow the in vivo visualisation of different immune cells activity in different diseases.

## CONFLICT OF INTEREST STATEMENT

The authors declare no competing interests.
